# A Trendline and Predictive Analysis of the First-Wave COVID-19 Infections in Malta

**DOI:** 10.3390/epidemiologia4010003

**Published:** 2023-01-10

**Authors:** Mitchell G. Borg, Michael A. Borg

**Affiliations:** 1 Department of Mechanical Engineering, Faculty of Engineering, University of Malta, MSD 2080 Msida, Malta; 2 Department of Naval Architecture, Ocean, and Marine Engineering, University of Strathclyde, Glasgow G4 0LZ, UK; 3 Infection Control Department, Mater Dei Hospital, MSD 2090 Msida, Malta; 4 Department of Microbiology, Faculty of Medicine and Surgery, University of Malta, MSD 2080 Msida, Malta

**Keywords:** COVID-19, Malta, trendline analysis, forecasting analysis, Weibull distribution

## Abstract

Following the first COVID-19 infected cases, Malta rapidly imposed strict lockdown measures, including restrictions on international travel, together with national social distancing measures, such as prohibition of public gatherings and closure of workplaces. The study aimed to elucidate the effect of the intervention and relaxation of the social distancing measures upon the infection rate by means of a trendline analysis of the daily case data. In addition, the study derived a predictive model by fitting historical data of the SARS-CoV-2 positive cases within a two-parameter Weibull distribution, whilst incorporating swab-testing rates, to forecast the infection rate at minute computational expense. The trendline analysis portrayed the wave of infection to fit within a tri-phasic pattern, where the primary phase was imposed with social measure interventions. Following the relaxation of public measures, the two latter phases transpired, where the two peaks resolved without further escalation of national measures. The derived forecasting model attained accurate predictions of the daily infected cases, attaining a high goodness-of-fit, utilising uncensored government-official infection-rate and swabbing-rate data within the first COVID-19 wave in Malta.

## 1. Introduction

Upon the identification of a novel coronavirus in China in January 2020, infections caused by the acute respiratory syndrome coronavirus 2 (SARS-CoV-2) rapidly spread worldwide [1]. Coronavirus disease 2019 (COVID-19) cases have been reported to be highly infectious and carry substantial morbidity and mortality [2]. Despite precautionary measures promoted by societal institutions, the impact of COVID-19 on healthcare systems has been significant, overwhelming hospital bed capacity and intensive care resources [3,4,5]. In an effort to ‘flatten the curve’, governments have implemented varying public health measures, which, at the most extreme, have involved lockdowns of large cities and countries [6,7]. These measures, however, have incurred substantial costs, and required large-scale logistical efforts to achieve an effective balance between the protection of human health and the national economy [8]. Studies have indicated that control measures have been effective [9,10], with a full lockdown imposition being the most effectual manner to mitigate the spread of the virus [11]. Yet, as the categories of control measures imposed by a government may vary substantially, very distinct cohorts of the population can truly be affected. As a result, analysing the virus spread in relation to discretised imposed measures has been a constraint in epidemiological analyses [12,13].

In order to constitute data-driven decisions, forecasting models by means of distribution fitting are commonly utilised to implement interventions within speculated timespans. These models have provided quantitative infection case projections that allow policymakers to plan appropriate interventions [14]. Of particular importance is short-term hospital resource allocation, such as emergency expansion and adoption of patient beds, hospital wards, or Nightingale hospitals, patient distributions within critical and intensive units, and diversion/suspension of routine hospital activities to permit functionality within rapidly changing trends. In turn, this endeavour requires predictive modelling that is ideally achievable without the need for specialised expertise or computational infrastructure, such that medical professionals may directly attain the data required for decision-making [15]. Specific enforcement measures must be historically justified to be effective on a national scale, yet epidemiological studies have not been able to isolate such measures due to external imported cases.

Dahal et al. [16] investigated transmission dynamics of COVID-19 at national and regional levels by using laboratory-confirmed RT-PCR data. Utilising dynamic phenomenological growth models, 10- and 20-day sequential forecasts of reproduction numbers were performed at weekly intervals over a timespan of two months (March to May 2021). The forecast indicated a declining trend of COVID-19 cases in Nepal, with a linear trajectory of COVID-19 incidence during the first wave and a sub-exponential growth pattern in the second. The study highlighted how computationally light mathematical models can provide reliable short-term projections and public health planning in such epidemic situations. Using similar techniques, Pajuelo-Reyes et al. [17] described the transmission of COVID-19 in the Amazonas region of Peru between March and July 2020.

Olumoyin et al. [18] argued that mitigation measures may impact epidemiological models with constant parameters. As a result, an algorithm was introduced to estimate the time-varying transmission rate for the COVID-19 pandemic in a background of different mitigation measures. Utilising cumulative and daily reported symptomatic infection numbers, the effect on the basic reproduction number of non-pharmaceutical mitigation measures, such as early detection of positive cases, subsequent contact tracing, and implementation of social distancing, was simulated. Using error metrics, the accuracy of the proposed algorithm was demonstrated for Italy, South Korea, the United Kingdom, and the United States.

The epidemiology of COVID-19 was also studied by Fonseca et al. [19] in the state of Sergipe, Brazil. The authors showed a positive correlation between the number of cases and deaths with demographic density. Using social parameters affecting the COVID-19 pandemic in Brazil, Marinho et al. [20] developed models that could be utilised by public managers for effective decision-making.

Ogwara et al. [21] observed the time-varying reproduction number of SARS-CoV-2 in Georgia, USA as state and countywide policies were implemented that enforced and relaxed COVID-19 public health measures. Policy changes were associated with increases or decreases at different time points. The reproduction number was seen to increase following the reopening of schools for in-person instruction. Additionally, Fain and Dobrovolny [22] developed a correlation model for SARS-CoV-2 infections using a complex partial differential equation mathematical model. The model generated dose–response curves for peak viral load, time of viral peak, viral growth rate, infection duration, and area under the viral curve.

Albeit the various studies conducted to establish and forecast the spread of COVID-19 have been impactful, the analyses have either had limitations in establishing the virus spread causation within a geographic area due to imported cases or incomplete data gathering, or had computationally intensive methodologies in forecasting the virus spread, hence requiring high-end computing architecture and highly trained analysts. In contrast, the primary aim of this work, in recognition of the limited imported cases as a result of the international travel ban, was to elucidate the effect of the distinct enforcement and relaxation social distancing measures upon the virus infection rate within the Maltese population by means of a historical trendline-fitting analysis. Additionally, as short-term hospital resource allocation and adaptation of routine hospital activities was critical throughout the pandemic, the secondary aim was developing a computationally efficient, yet accurate, forecasting model for the COVID-19 infection spread, using SARS-CoV-2 positive-case and swab-testing data in Malta, to be solved on commonly utilised office machines and spreadsheet software for data-driven logistics insights and decision-making. 

Malta is a Mediterranean island country with a population of approximately 500,000 inhabitants. Its first SARS-CoV-2 isolate was reported on 7 March 2020. Following the initial case, the country experienced an epidemic until the end of July, with a peak in mid-April, in what was the first wave of COVID-19 in the country. Various interventions were introduced by the government to address the challenge, with international and national travel bans being the most substantial. A fundamental component of the national strategy was an exhaustive track-and-trace strategy, which reached a frequency of almost 2000 swabs per 100,000 residents per week by the end of the first wave.

## 2. Methodologies

### 2.1. Trendline Analysis

#### 2.1.1. Data Collection

The daily number of new COVID-19 cases, together with the respective number of swab-tests taken, were retrieved from the official website of the Ministry of Health in Malta issued from 21 February 2020 onwards. The key interventions introduced by the Maltese government throughout the first wave were also identified, together with the dates when these interventions were implemented and discontinued. These are summarised in Table 1. It should be noted that, as foreign and local travel had ceased within the island, together with an effective track-and-trace practice, the virus spread data were deemed to be solely related to national cases.

#### 2.1.2. Trendline Functions

In an effort to establish the trend by which the cumulative number of infections varied over the first pandemic wave in Malta, a trendline analysis was implemented. Primarily, a linear function (Equation (1)), was utilised upon the case data. Subsequently, logistic functions, based upon the cumulative distribution functions of the Exponential distribution (Equation (2)) and the Weibull distribution (Equations (3)–(5)), were implemented. The Weibull-based logistic function was developed further such that three distinct functions were coupled, establishing a multi-logistic function.

(1) $$N_{c}=M_{c1}n$$

(2) $$N_{c}=M_{c1}\cdot \left(1-e^{-\left(\frac{n}{\lambda_{c1}}\right)}\right)$$

(3) $$N_{c}=M_{c1}\cdot \left(1-e^{-{\left(\frac{n}{\lambda_{c1}}\right)}^{k_{c1}}}\right)$$

(4) $$N_{c}=M_{c1}\cdot \left(1-e^{-{\left(\frac{n}{\lambda_{c1}}\right)}^{k_{c1}}}\right)+M_{c2}\cdot \left(1-e^{-{\left(\frac{n}{\lambda_{c2}}\right)}^{k_{c2}}}\right)$$

(5) $$N_{c}=M_{c1}\cdot \left(1-e^{-{\left(\frac{n}{\lambda_{c1}}\right)}^{k_{c1}}}\right)+M_{c2}\cdot \left(1-e^{-{\left(\frac{n}{\lambda_{c2}}\right)}^{k_{c2}}}\right)+M_{c3}\cdot \left(1-e^{-{\left(\frac{n}{\lambda_{c3}}\right)}^{k_{c3}}}\right)$$

where $N_{c}$ is the cumulative number of positively diagnosed cases, n is the day number, $\;M_{c1},\;\;M_{c2},\;\;M_{c3}$ are the case-dependent magnitude parameters, $\lambda_{c1},\;\;\lambda_{c2},\;\;\lambda_{c3}$ are the case-dependent scale parameters, and $k_{c1},\;k_{c2},\;\;k_{c3}$ are the case-dependent shape parameters.

In relation to the cumulative infected cases, a daily infected case trendline analysis was undertaken by implementing the arithmetic time-derivative of the cumulative functions (Equations (6)–(10)).

(6) $$\dot{N_{c}}=M_{c1}$$

(7) $$\dot{N_{c}}=\frac{M_{c1}}{\lambda_{c1}}\cdot \left(e^{-\left(\frac{n}{\lambda_{c1}}\right)}\right)$$

(8) $$\dot{N_{c}}=\frac{M_{c1}\cdot k_{c1}\cdot n^{k_{c1}-1}}{{\left(\lambda_{c1}\right)}^{k_{c1}}}\cdot \left(e^{-{\left(\frac{n}{\lambda_{c1}}\right)}^{k_{c1}}}\right)$$

(9) $$\dot{N_{c}}=\frac{M_{c1}\cdot k_{c1}\cdot n^{k_{c1}-1}}{{\left(\lambda_{c1}\right)}^{k_{c1}}}\cdot \left(e^{-{\left(\frac{n}{\lambda_{c1}}\right)}^{k_{c1}}}\right)+\frac{M_{c2}\cdot k_{c2}\cdot n^{k_{c2}-1}}{{\left(\lambda_{c2}\right)}^{k_{c2}}}\cdot \left(e^{-{\left(\frac{n}{\lambda_{c2}}\right)}^{k_{c2}}}\right)$$

(10) $$\dot{N_{c}}=\frac{M_{c1}\cdot k_{c1}\cdot n^{k_{c1}-1}}{{\left(\lambda_{c1}\right)}^{k_{c1}}}\cdot \left(e^{-{\left(\frac{n}{\lambda_{c1}}\right)}^{k_{c1}}}\right)+\frac{M_{c2}\cdot k_{c2}\cdot n^{k_{c2}-1}}{{\left(\lambda_{c2}\right)}^{k_{c2}}}\cdot \left(e^{-{\left(\frac{n}{\lambda_{c2}}\right)}^{k_{c2}}}\right)\;+\frac{M_{c3}\cdot k_{c3}\cdot n^{k_{c3}-1}}{{\left(\lambda_{c3}\right)}^{k_{c3}}}\cdot \left(e^{-{\left(\frac{n}{\lambda_{c3}}\right)}^{k_{c3}}}\right)$$

where $\dot{N_{c}}$ is the number of positive cases per day.

In a similar manner, the cumulative number of swab-tests over the first wave was analysed by utilising a linear function (Equation (11)) and a Weibull-based logistic function (Equation (12)).

(11) $$N_{s}=M_{s1}\cdot \left(n+14\right)$$

(12) $$N_{s}=M_{s1}\cdot \left(1-e^{-{\left(\frac{n+14}{\lambda_{s1}}\right)}^{k_{s1}}}\right)$$

where $N_{s}$ is the cumulative number of swab-tests, $\;M_{s1}$ is the swab-test-dependent magnitude parameter, $\lambda_{s1}$ is the swab-test-dependent scale parameter, and $k_{s1}$ is the swab-test-dependent shape parameter. 

In relation to the cumulative swab-test number, a daily swab-test trendline analysis was undertaken by implementing the arithmetic time-derivative of the cumulative functions (Equations (13) and (14)).

(13) $$\dot{N_{s}}=M_{s1}$$

(14) $$\dot{N_{s}}=\frac{M_{s1}\cdot k_{s1}\cdot {\left(n+14\right)}^{k_{s1}-1}}{{\left(\lambda_{s1}\right)}^{k_{s1}}}\cdot \left(e^{-{\left(\frac{n+14}{\lambda_{s1}}\right)}^{k_{s1}}}\right)$$

where $\dot{N_{s}}$ is the number of swab-tests per day.

### 2.2. Predictive Analysis

A prediction model was derived by utilising a logarithmic growth rate equation for the daily diagnosed cases (Equation (15)).

(15) $$K_{c_{n}}=\frac{\Delta \ln N_{c_{n}}}{\Delta n}=\ln\left(N_{c_{n}}\right)-\ln\left(N_{c_{n-1}}\right)$$

where $N_{c_{n}}$ is the cumulative number of positively diagnosed cases on a given day, n is the considered day, and $K_{c_{n}}$ is the daily positive-case logarithmic growth rate. 

From the formula, the necessary model output was $N_{c_{n}}$, and hence, $K_{c_{n}}$ was required to be determined. To statistically predict $K_{c_{n}}$, a two-parameter Weibull distribution fit was implemented upon the logarithmic growth rate data of the previous days to establish the Weibull scale and shape parameters. The parameters were rolling parameters as the process was done daily throughout the time period. The scale and shape parameters were then utilised within an inverse cumulative distribution in the form of a quantile function for the Weibull distribution to establish a $K_{c_{n}}$ range (Equation (16)).

(16) $$K_{c_{n}}=Q\left(p;\lambda_{c};k_{c}\right)=\lambda_{c}\sqrt[{k_{c}}]{-\ln\left(1-F\left(K_{c_{n}}\right)\right)}=\lambda_{c}\sqrt[{k_{c}}]{-\ln\left(1-p\right)}$$

where p is the occurrence probability, $\lambda_{c}$ is the case-dependent Weibull scale parameter, and $k_{c}$ is the case-dependent Weibull shape parameter.

In addition, to account for the variation in daily swab-testing, and overcome the assumption of a constant swab-testing-rate, a swab-test factor ($c_{s}$) was introduced and coupled with $K_{c_{n}}$ (Equation (17)).

(17) $$N_{c_{n}}=\exp\left(\ln\left(N_{c_{n-1}}\right)+c_{s}K_{c_{n}}\right)$$

The swab-test coefficient was established to be the ratio between the swab-test logarithmic growth rate on day n and the average swab-test logarithmic growth rate of the considered prior days (Equation (18)).

(18) $$c_{s}=\frac{K_{s_{n}}}{\frac{1}{t}\cdot \sum_{i=n-t}^{n-1}K_{s_{i}}}=\frac{\ln\left(N_{s_{n}}\right)-\ln\left(N_{s_{n-1}}\right)}{\frac{1}{t}\cdot \sum_{i=n-t}^{n-1}\left(\ln\left(N_{s_{i}}\right)-\ln\left(N_{s_{i-1}}\right)\right)}$$

where $K_{s_{n}}$ is the swab-test daily logarithmic growth rate, t is the number of considered prior days, and $N_{s_{n}}$ is the cumulative number of swab-tests on a given day. 

By incorporating the prior equations, the predictive model was derived to establish the daily number of infected cases over a time period (Equations (19)–(21)).

(19) $$N_{c_{n}}=\exp\left(\ln\left(N_{c_{n-1}}\right)+c_{s}\cdot \left(\lambda_{c}\sqrt[{k_{c}}]{-\ln\left(1-p\right)}\right)\right)$$

(20) $$N_{c_{n}}=\exp\left(\ln\left(N_{c_{n-1}}\right)+\frac{K_{s_{n}}}{\frac{1}{t}\cdot \sum_{i=n-t}^{n-1}K_{s_{i}}}\cdot \lambda_{c}\sqrt[{k_{c}}]{-\ln\left(1-p\right)}\right)$$

(21) $$N_{c_{n}}=N_{c_{n-1}}\cdot \exp\left(\frac{K_{s}{}_{n}}{\frac{1}{t}\cdot \sum_{i=n-t}^{n-1}K_{s}{}_{i}}\cdot \lambda_{c}\sqrt[{k_{c}}]{-\ln\left(1-p\right)}\right)$$

## 3. Results

### 3.1. Trendline Analysis

#### 3.1.1. Positive Infected-Case Function

**Figure 1:**
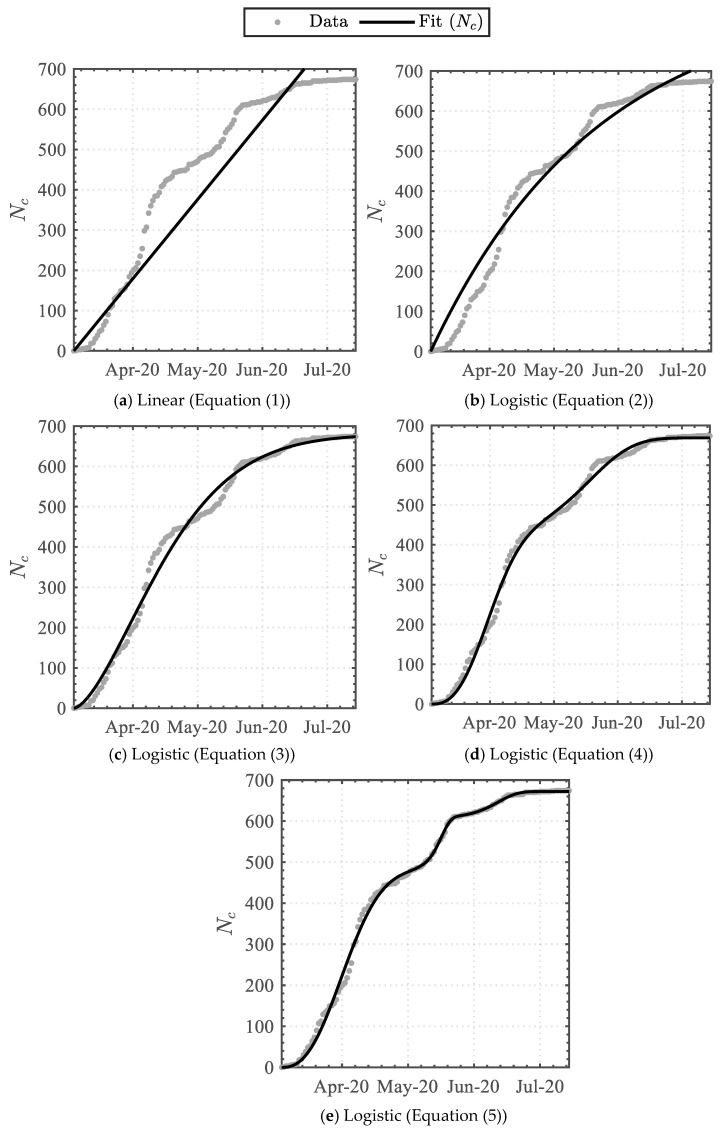
Trendline analysis of the cumulative infected cases.

Applying Equations (1)–(5) to the cumulative dataset attained the data-driven variables detailed in Table 2. The linear function attained the least similarity, whereas the triple Weibull-based logistic function attained the highest similarity, with coefficients of determination (R^2^) of 0.860 and 0.998, respectively. The functions were graphically superimposed upon the dataset, as illustrated in Figure 1.

**Figure 2:**
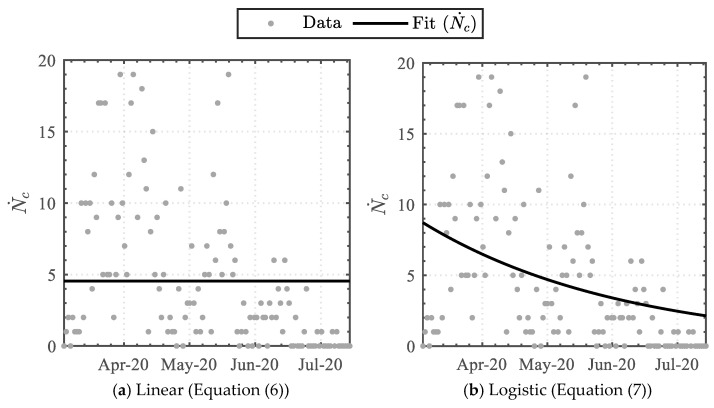

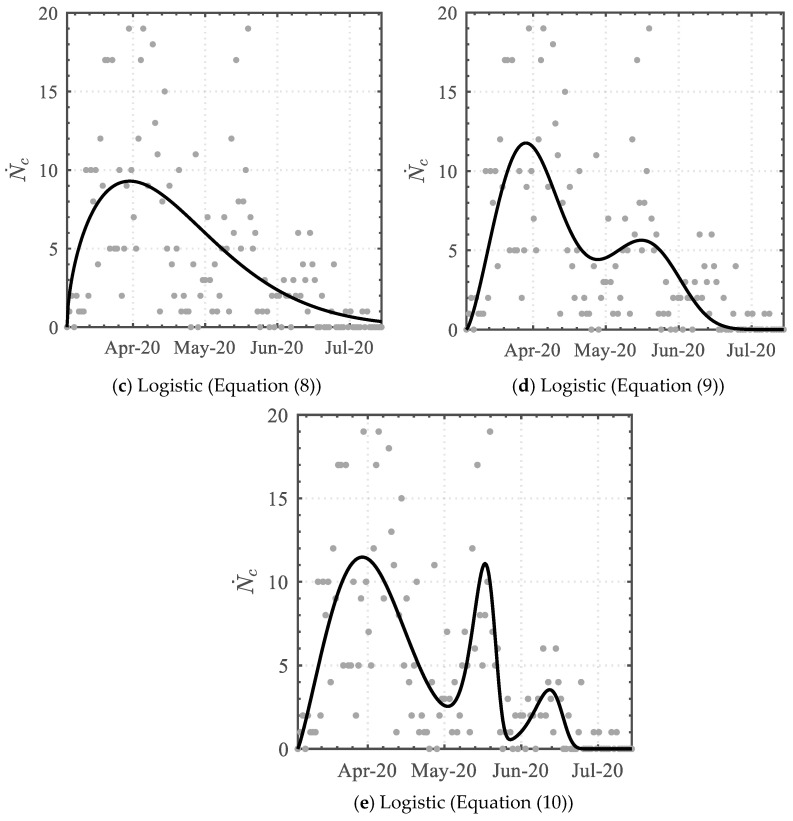
Trendline analysis of the daily infected cases.

**Figure 3:**
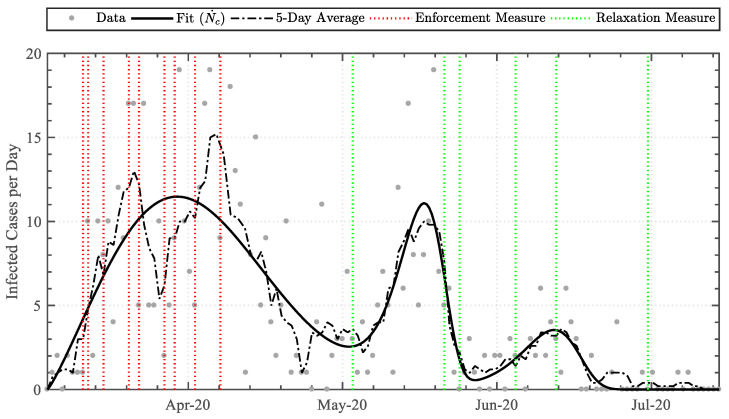
Comparison between the derived trendline fit (Equation (10)) and the 5-day average, together with the intervention enforcement/relaxation dates.

Implementing Equations (6)–(10) to the daily infected cases dataset attained the data-driven variables detailed in Table 3. The linear-derivative function attained the least similarity, whereas the triple Weibull-based logistic-derivative function attained the highest similarity, with coefficients of determination ($R^{2}$) of 0.0 and 0.566, respectively. The functions were graphically superimposed upon the dataset, as illustrated in Figure 2. Furthermore, as the triple Weibull-based logistic-derivative function attained the highest similarity, portraying a high correspondence, the key dates on which the nationally imposed measures were enforced or relaxed (see Table 1) were additionally incorporated, as illustrated in Figure 3.

#### 3.1.2. Swab-Test Function

**Figure 4:**
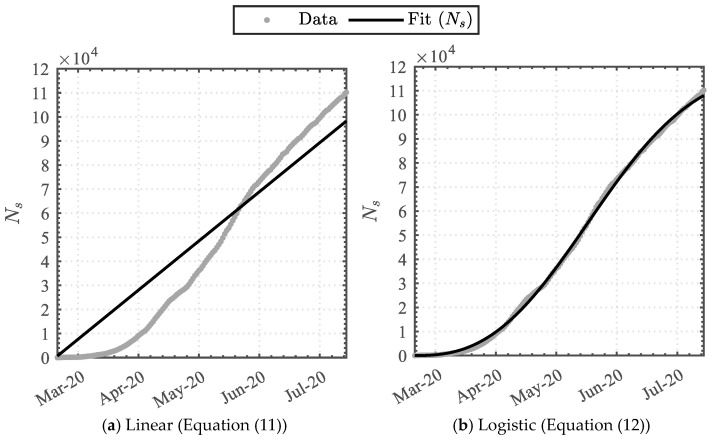
Trendline analysis of the cumulative swab-tests.

Applying Equations (11) and (12) to the cumulative swab dataset attained the data-driven variables detailed in Table 4. The linear function attained the least similarity, whereas the Weibull-based logistic function attained the highest similarity, with coefficients of determination ($R^{2}$) of 0.901 and 0.999, respectively. The functions were graphically superimposed upon the dataset, as illustrated in Figure 4.

**Figure 5:**
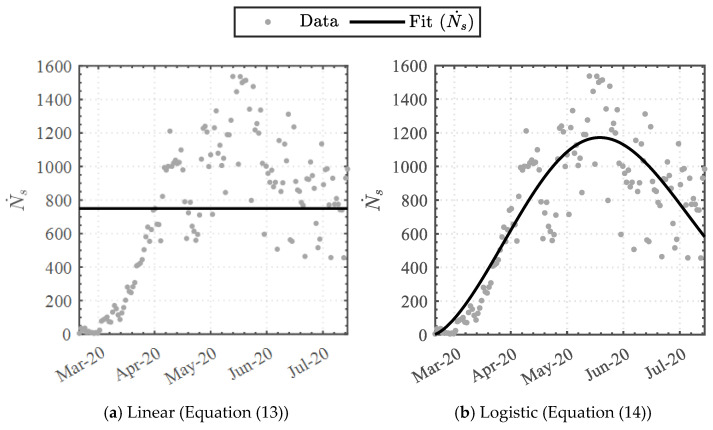
Trendline analysis of the daily swab-tests.

**Figure 6:**
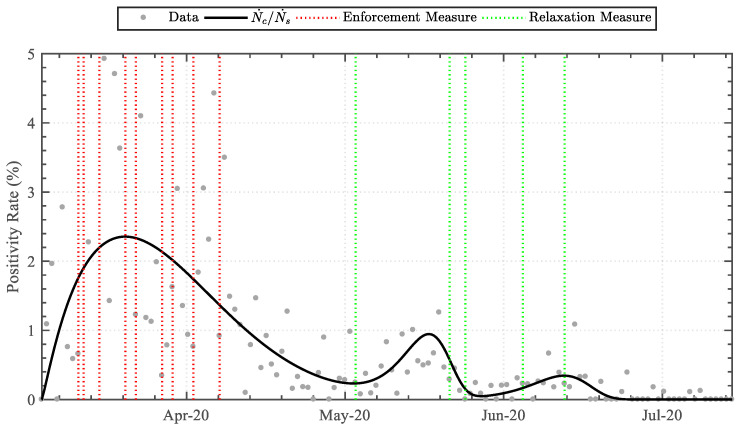
Positivity rate throughout the first wave.

Implementing Equations (13) and (14) to the daily swab count dataset attained the data-driven variables detailed in Table 5. The linear-derivative function attained the least similarity, whereas the Weibull-based logistic-derivative function attained the highest similarity, with coefficients of determination ($R^{2}$) of 0.0 and 0.762, respectively. The functions were graphically superimposed upon the dataset, as illustrated in Figure 5. Furthermore, the infection-rate positivity ratio was established by coupling Equations (10) and (14), illustrated in Figure 6. The key dates on which the nationally imposed measures were enforced or relaxed (see Table 1) were additionally incorporated.

### 3.2. Predictive Analysis

**Figure 7:**
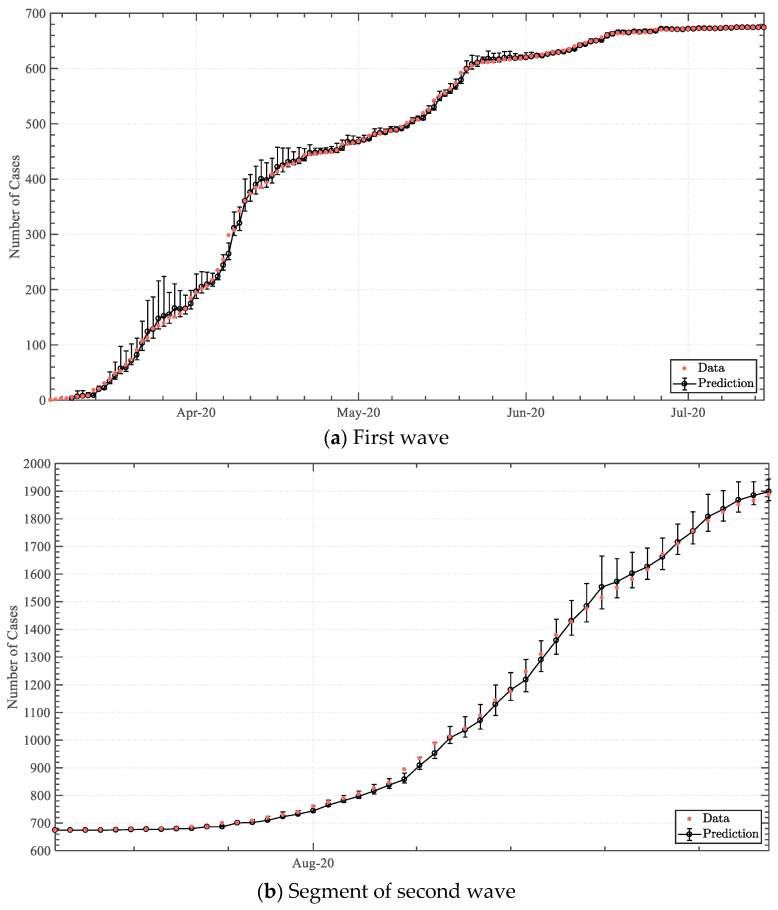
One-day predictive output of infected cases within the first & second COVID-19 waves.

**Figure A1:**
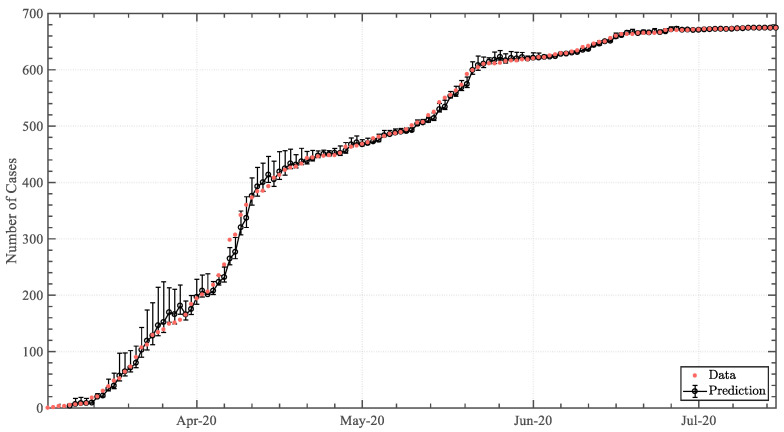
Two-day infected-case predictive output (first wave).

**Figure A2:**
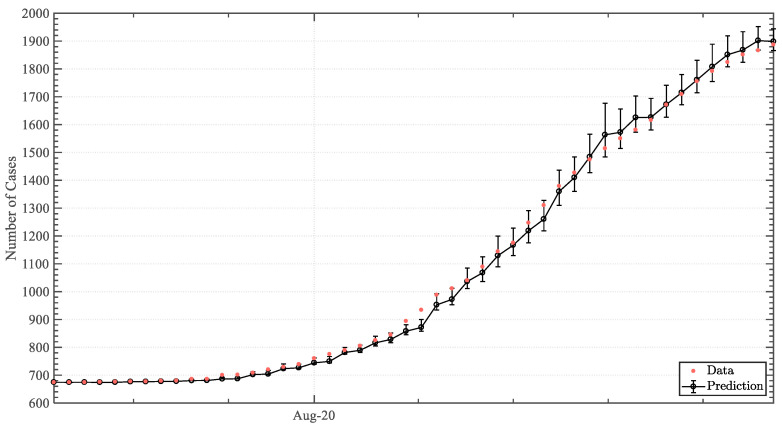
Two-day infected-case predictive output (second wave segment).

**Figure A3:**
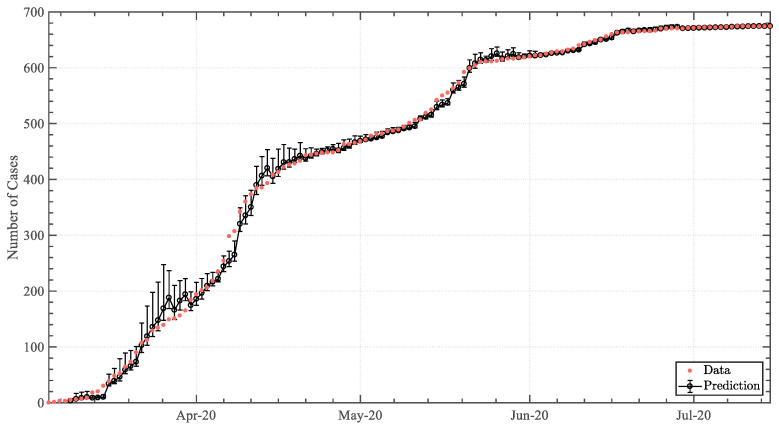
Three-day infected-case predictive output (first wave).

**Figure A4:**
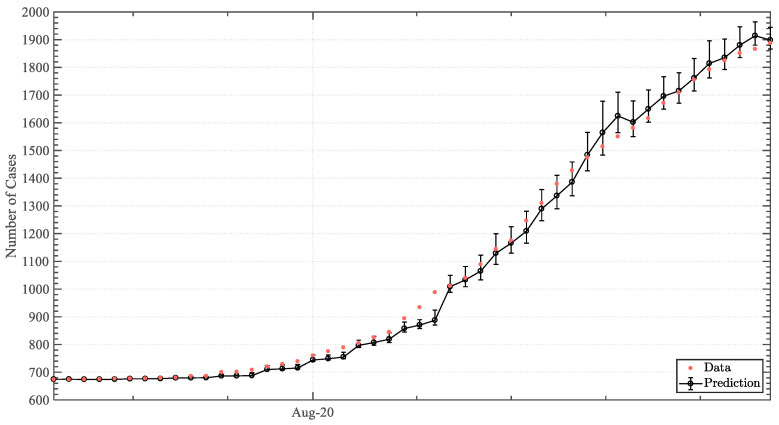
Three-day infected-case predictive output (second wave segment).

**Figure A5:**
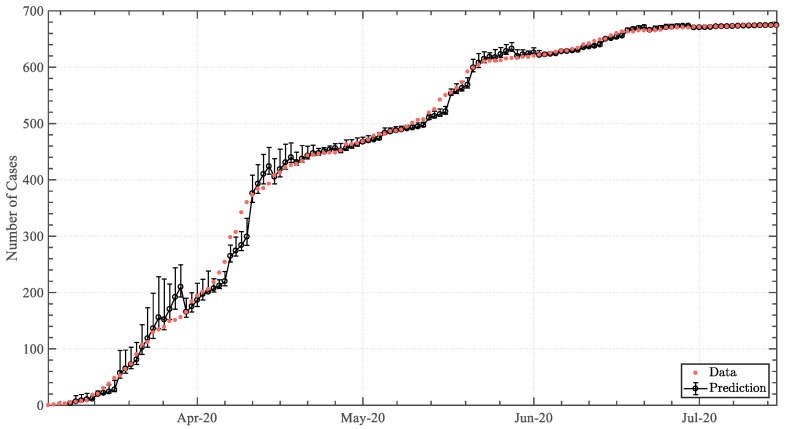
Four-day infected-case predictive output (first wave).

**Figure A6:**
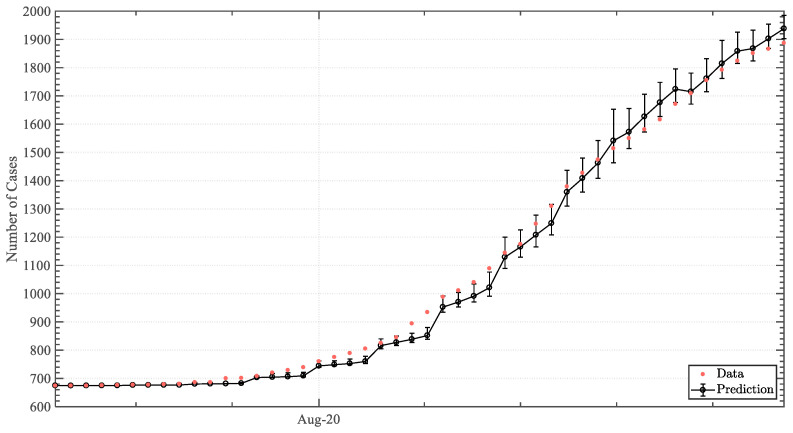
Four-day infected-case predictive output (second wave segment).

**Figure A7:**
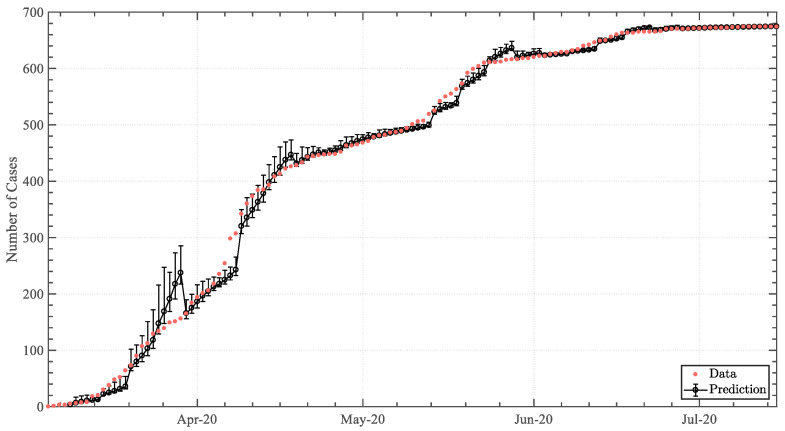
Five-day infected-case predictive output (first wave).

**Figure A8:**
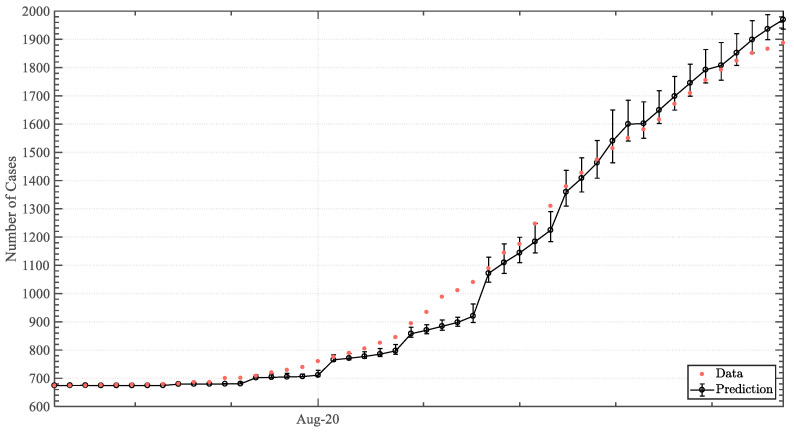
Five-day infected-case predictive output (second wave segment).

The forecasting model was applied for the entire first wave (7 March–15 July 2020), and a portion of the second wave (16 July–31 August 2020), to establish the continuity capacity of the model. Employed within a one-day (Figure 7) to a five-day (Figure A1, Figure A2, Figure A3, Figure A4, Figure A5, Figure A6, Figure A7 and Figure A8) prediction framework, the statistical model attained good agreement with the dataset, achieving a global coefficient of determination ($R^{2}$) of 0.9995 to 0.9955 between the statistical model median outputs and the actual dataset. Good agreement was also attained for predictions beyond five days. Particularly for the one-day, two-day, and three-day forecasting, solely 4.5%, 7.8%, and 12.3% of the data-points fell outside of the 0th–95th percentile prediction band, respectively. The explicit statistical modelling methodology was therefore deemed to have been validated to a high degree of accuracy.

## 4. Discussion

Implementing a trendline analysis upon the daily case dataset, as opposed to a sole moving average, permitted superimposing the dates of enforcement and relaxation measures to qualitatively shed light on the effect of the measures along the trend [23]. The infection rate trend increased exponentially throughout the initial days of the pandemic, prior to social measures. Upon the implementation of enforcement measures related to public transport and closure of workplaces, sports facilities, law courts, religious places, and service outlets, the rate-of-change of the daily cases diminished. This acknowledged the effect of the social-distancing enforcement measures, inhibiting the spread of the virus. The subsequent enforcement measures related to the prohibition of public gatherings, and closure of education establishments and non-essential retail/service outlets further decreased the rate-of-change steadily, attaining a peak average infection rate of 11.5 cases per day on 31 March.

The relaxation of public transport measures, re-opening of non-essential retail outlets, and increased size of public gatherings (to a maximum of three persons) on 4 May resulted in the second phase, occurring with an exponential increase in infection rate. The rate, however, peaked rapidly and diminished to a low value within a short timeframe of six days. The third phase was evident following relaxation measures of public gatherings (to a maximum of four persons) and re-opening of service outlets and public places on 22 May. This had a lower rate-of-change than the second phase and rapidly levelled off by 15 July. 

The initiation of the second and third phases was consistent with the relaxation of public gathering social distancing measures [24]. As higher numbers of persons were permitted to be in close contact, the possibility of virus transmission increased. Nevertheless, the second and third peaks were much lower and a substantial drop in infection-rate number followed with no new enforcement measures implemented. The reason for this may be attributed to societal diligence in re-orienting itself to effective social distancing [25]. The wearing of masks or visors in shops and on public transport had become obligatory by law on 4 May and may have contributed to the achievement of control in the second and third peaks [26]. A more likely hypothesis, however, may be the effectiveness of test-and-trace that, by this time, had been expanded and consolidated [27]. Approximately 110,000 swab-tests were performed within the first wave, where the peak median swab-tests per day was found to be approximately 1200 on 18 May 2020. Along the second phase time-period, the mean swabbing frequency had increased to over 9000 swabs per week, with peak median positivity ratios of 2.34, 0.95, and 0.34 cases per hundred swabs identified within the first, second, and third phases, respectively. Every positive case was isolated within 24 h of testing with concurrent quarantine of significant contacts. This was possible given the low positivity rate during the second and third phases, which was below 1%. By means of the trendline, it may be argued that, as testing was increasing, had the relaxation measure of 4 May been implemented two weeks later, it would have allowed the infection-rate to level off. Accordingly, the relaxation measure of 1 July would potentially have been implemented earlier.

With regard to the forecasting model, the derivation of an explicit statistical model based upon the logarithmic growth rate was found to be an accurate and computationally feasible methodology, achieving a global $R^{2}$ of over 0.99 and a total computation time of less than five seconds on a typical office machine. This methodology attained the logical implementation of solving for the accumulative number of cases on a particular day ($N_{n}$) by determining the logarithmic growth rate ($K_{n}$) via a statistical analysis utilising an inverse cumulative distribution (quantile) function based on a Weibull distribution, together with incorporating a swab-test coefficient ($c_{n}$) to account for the correlation between tests undertaken and positively infected cases. 

Incorporating a Weibull distribution fit was advantageous due to its adaptability, permitting the comprehension of both symmetric and non-symmetric distributions whilst interpolating between the exponential distribution and the Rayleigh distribution via a two-parameter implementation. The Weibull cumulative distribution function put forward an explicit function, encompassing two parameters that can be estimated from a dataset, and hence efficiently solvable, in contrast to the application of implicit formulation functionality [28]. This aspect was deemed imperative as high-end statistical approaches tend to lie beyond the statistical knowledge-capacity of medical professionals and the processing power of office machines and commonly utilised spreadsheet software [29,30]. In addition, this model permitted the utilisation of historical data of positive cases and swab-tests, rather than implementing trends that disregard swab-testing correlation. This model may distinctively be utilised for short-to-medium term quantitative risk assessments. Furthermore, week-long facility logistic decisions, such as the number of beds in emergency wards, ventilators, and on-call staff personnel, may be substantiated utilising the model.

By collating and discussing the different statistical modelling and prediction techniques for COVID-19, Yadav and Akhter [31] pinpointed the significance of utilising a single distribution to fit and represent the true virus spread, such that effective data-driven policies may be made. The implemented two-parameter Weibull distribution methodology implemented within this work succeeded in achieving this critical indication. The model, however, is limited by the diminishing capacity of the distribution if left-censored data are utilised [32,33]. Moreover, when furthering the forecasting to a larger timespan, the deviation from the true result may increase substantially as a result of the fundamental approach [34]. Nonetheless, Weibull analyses are typically utilised for medical statistics as the methodology has been acknowledged to sustain accuracy despite an extremely small dataset [35]. In fact, within the context of SARS-CoV-2 statistical analyses, this approach has been notably applied to establish the incubation period of the virus [36,37]. As a result, a rudimentary yet accurate forecasting model was established, encompassing the imperative capacity of accurately predicting positive cases within the termination of one wave and the initiation of another.

## 5. Conclusions

This study presented a novel statistical model, incorporating swab-testing rates coupled with Weibull-distributed historical data of the SARS-CoV-2 positive-case logarithmic growth rate, to predict the virus infection rate and establish an accurate projection through a numerically explicit framework. The model was validated utilising infection rate data within Malta. Furthermore, an epidemiological elaboration of infection trends was established utilising trendline analyses for the purpose of evaluating social distancing enforcement and relaxation measures upon the virus spread within the population. 

## Figures and Tables

**Table 1:** Intervention enforcement/relaxation measures & dates.

(**A**) **Lockdown Measures**	**Enforcement Date**	**Relaxation Date**
International travel ban	21 March 2020	1 July 2020
Lockdown of vulnerable persons	28 March 2020	5 June 2020
National travel ban (essential travel only between the Maltese islands)	3 April 2020	4 May 2020
(**B**) **Social Distancing Measures**	**Enforcement Date**	**Relaxation Date**
Public transport measures (daily decontamination; passenger screening; standing passengers disallowed; windows open; air-conditioning system off; no monetary change)	12 March 2020	4 May 2020
Closure of workplaces and distancing of workers	13 March 2020	5 June 2020
Closure of sports facilities	13 March 2020	5 June 2020
Closure of law courts and local tribunals	13 March 2020	5 June 2020
Closure of religious places	13 March 2020	13 June 2020
Closure of service outlets and public places	16 March 2020	22 May 2020
Closure of education establishments	21 March 2020	5 June 2020
Closure of non-essential retail outlets	23 March 2020	4 May 2020
Closure of non-essential service outlets	23 March 2020	22 May 2020
Measures to protect elderly and high-risk groups	28 March 2020	5 June 2020
Prohibition of public gatherings (limits of 3 persons, 4 persons, and 6 persons)	30 March 2020	4 May 2020, 22 May 2020, 5 June 2020
Suspension of visits to homes for the elderly and the national hospital	8 April 2020	25 May 2020

**Table 2:** Cumulative infected cases trendline data.

Equation	$M_{c1}$	$\lambda_{c1}$	$k_{c1}$	$M_{c2}$	$\lambda_{c2}$	$k_{c2}$	$M_{c3}$	$\lambda_{c3}$	$k_{c3}$	$R^{2}$
Equation (1)	6.486	-	-	-	-	-	-	-	-	0.806
95% CI	6.677	-	-	-	-	-	-	-	-	
	6.296									
Equation (2)	885.6	78.32	-	-	-	-	-	-	-	0.970
95% CI	940.3	87.12	-	-	-	-	-	-	-	
	830.9	69.53								
Equation (3)	680.0	49.61	1.581	-	-	-	-	-	-	0.991
95% CI	691.2	51.01	1.655	-	-	-	-	-	-	
	668.9	48.21	1.508							
Equation (4)	434.8	31.38	2.628	234.3	79.05	4.687	-	-	-	0.996
95% CI	472.0	33.12	2.836	273.8	82.32	5.888	-	-	-	
	397.6	29.64	2.419	194.7	75.78	3.486				
Equation (5)	484.6	33.64	2.486	64.03	100.2	12.25	123.4	73.59	17.98	0.998
95% CI	495.6	34.41	2.606	82.83	103.7	20.26	145.1	74.73	23.93	
	473.6	32.86	2.367	45.23	96.69	4.327	101.7	72.45	12.03	

**Table 3:** Daily infected cases trendline data.

	$M_{c1}$	$\lambda_{c1}$	$k_{c1}$	$M_{c2}$	$\lambda_{c2}$	$k_{c2}$	$M_{c3}$	$\lambda_{c3}$	$k_{c3}$	$R^{2}$
Equation (6)	4.542	-	-	-	-	-	-	-	-	0.0
95% CI	5.427	-	-	-	-	-	-	-	-	
	3.657									
Equation (7)	818.4	93.88	-	-	-	-	-	-	-	0.183
95% CI	1077	137.1	-	-	-	-	-	-	-	
	560.0	50.71								
Equation (8)	618.8	50.32	1.568	-	-	-	-	-	-	0.376
95% CI	716.3	57.79	1.809	-	-	-	-	-	-	
	521.3	42.84	1.327							
Equation (9)	371.1	30.77	2.357	222.4	76.42	5.118	-	-	-	0.459
95% CI	476.9	36.07	2.836	273.8	82.32	5.888	-	-	-	
	265.3	29.64	2.419	194.7	75.78	3.486				
Equation (10)	429.6	33.94	2.168	51.61	99.86	18.60	124.8	74.35	17.37	0.556
95% CI	495.6	34.41	2.606	82.83	103.7	20.26	145.1	74.73	23.93	
	473.6	32.86	2.367	45.23	96.69	4.327	101.7	72.45	12.03	

**Table 4:** Cumulative swab-test trendline data.

	$M_{s1}$	$\lambda_{s1}$	$k_{s1}$	$R^{2}$
Equation (11)	673.0	-	-	0.901
95% CI	650.1	-	-	
	695.9			
Equation (12)	118,770.3	104.9	2.652	0.999
95% CI	117,054.6	103.7	2.612	
	120,486.0	106.1	2.692	

**Table 5:** Daily swab-tests trendline data.

	$M_{s1}$	$\lambda_{s1}$	$k_{s1}$	$R^{2}$
Equation (13)	749.8	-	-	0.0
95% CI	678.8	-	-	
	820.9			
Equation (14)	127,657.8	110.0	2.499	0.762
95% CI	118,635.2	105.2	2.317	
	136,680.4	114.8	2.681	

## Data Availability

Not applicable.
